# Calicovesicostomy surgery in the patient with ureteral rupture

**DOI:** 10.1016/j.eucr.2022.102133

**Published:** 2022-06-18

**Authors:** Farzad Allameh, Seyyed Ali Hojjati, Saba Faraji, Amirhossein Eslami, Maryam Garousi

**Affiliations:** a Laser Application in Medical Sciences Research Center, Shohada-e-Tajrish Hospital, Shahid Beheshti University of Medical Sciences, Tehran, Iran; b Urology and Nephrology Resaerch Center, Shahid Beheshti University of Medical Sciences, Tehran, Iran; c Department of Psychiatry, Roozbeh Hospital, Tehran University of Medical Sciences, Tehran, Iran; d Department of Urology, Shohada-e-Tajrish Hospital, Shahid Beheshti University of Medical Sciences, Tehran, Iran; e Radiation Oncology Department, Iran University of Medical Sciences, Tehran, Iran

## Abstract

Transurethral lithotripsy (TUL) surgery, which is used to crush ureteral stones, can have complications, including bleeding, infection, ureteral stenosis, ureteral rupture, and ureteral avulsion. In this study, we present a 45-year-old woman who was referred, due to a right ureteral rupture during transurethral lithotripsy surgery. At first, the patient underwent ureteroneocystostomy surgery by a combination of boari flap and psoas hitch techniques. Due to the obstruction and necrosis at the anastomosis site, the patient underwent calicovesicostomy surgery.

## 1 Introduction

Transurethral lithotripsy (TUL) surgery, which is used to crush ureteral stones, can have complications, including bleeding, infection, ureteral stenosis, ureteral rupture, and ureteral avulsion.^1^

Ureteral rupture usually requires ureteroneocystostomy surgery or ureteral repair. Commonly used techniques include boari flap technique, psoas hitch technique, ureteroureterostomy, autotransplantation of kidney, and also interposition of ileum or jejunum. Depending on the location and extent of ureteral injury, the type of reconstructive surgery will vary.

The ureteral stent can be used if the ureteral trauma is minor, but it is not effective in case of major injuries and complete ureteral rupture or ureteral avulsion. In such cases, in the acute phase of trauma, a nephrostomy can be performed for the patient to maintain urine drainage from the kidney.^2^

### 1.1 Case report

The patient is a 45-year-old woman who was referred, due to a right ureteral rupture. The patient underwent TUL surgery due to a 10 mm stone in the proximal right ureter, which was ruptured during surgery. Her vital signs were stable. Abdominopelvic computed tomography (CT) scan without intravenous injection was done and a urine leak was seen around the kidney and right ureter.

At first, ureteroscopy was performed to insert a stent, which was not successful. The patient underwent midline laparatomy incision. During surgery, the kidney was first released from the surrounding tissues and descended. Then nephropexy was performed. Ureteroneocystostomy surgery was performed using a combination of boari flap and psoas hitch techniques and a total of about 20 cm of the ureteral defect was compensated. We shaped the flap from the posterior and dome, above the ureteral orifice on the opposite side, so that it is wide and the possibility of necrosis is low. A nephrostomy was inserted.

**Fig. 1:**
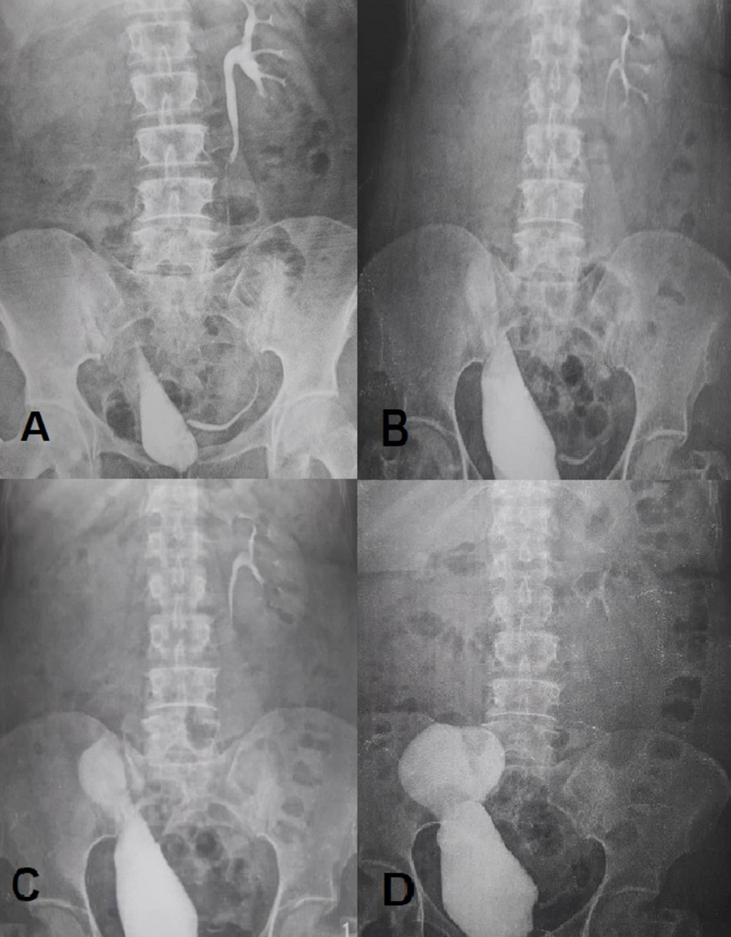
Intravenous Pyelogram (IVP) before calicovesicostomy surgery. The location of the ureteral re-implantation was not well contrasted. 15 minutes (A), 1 hour (B), 1.5 hour (C), 4 hour (D).

Eight months after surgery, the patient developed flank pain. An intravenous pyelogram (IVP) was performed for the patient (Fig. 1). In the nephrogram phase, the contrast agent was secreted from the right kidney with a delay, and the right kidney had hydronephrosis. In delayed stereotypes, the location of the ureteral re-implantation was not well contrasted. Stenosis and necrosis of the anastomosis site have been suggested. A nephrostomy was inserted in the right kidney and then, CT nephrostography was performed. The contrast material did not pass through the anastomosis site and did not enter the bladder and bladder capacity was acceptable.

**Fig. 2:**
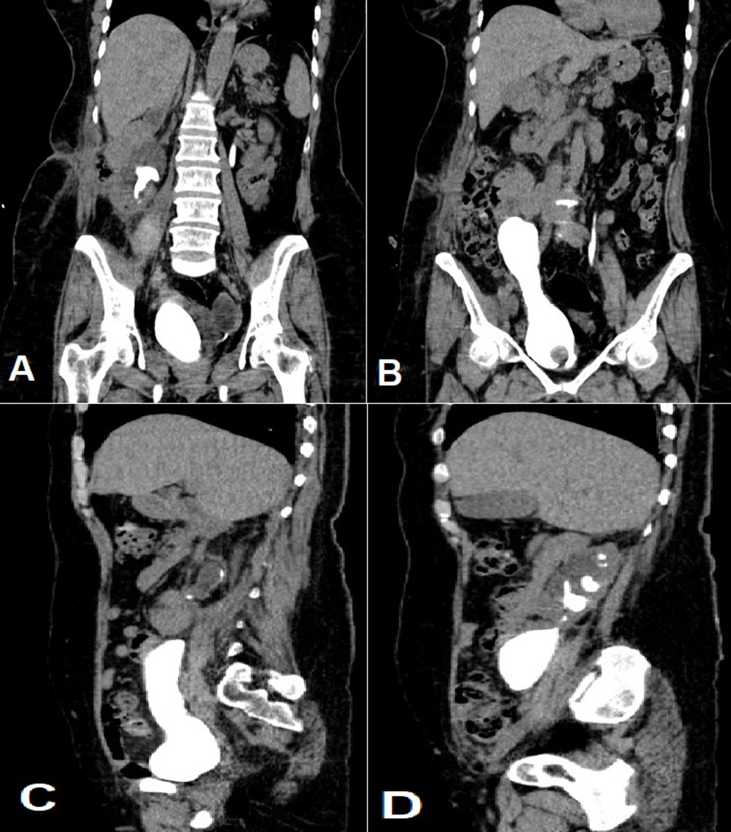
Computed Tomography (CT) cystography after Calicovesicostomy surgery. Anastomosis site was open and no stenosis or obstruction was seen. Coronal view (A–B), Sagittal view (C–D).

The patient underwent calicovesicostomy surgery, in which the bladder was anastomosed directly to the lower calyx. Due to the patient's condition and lack of proper response to the patient's initial surgery, the decision was made for this type of surgery. The day after the operation, nephrostography was performed in which there wasn't any contrast leakage and the contrast agent entered the bladder. After one month, the patient's stent was removed. In the patient's 2-month follow-up, CT cystography was performed in which the anastomosis site was open and no stenosis or obstruction was seen (Fig. 2). The patient had flank pain and frequency for a while, but then the patient's symptoms disappeared.

**Fig. 3:**
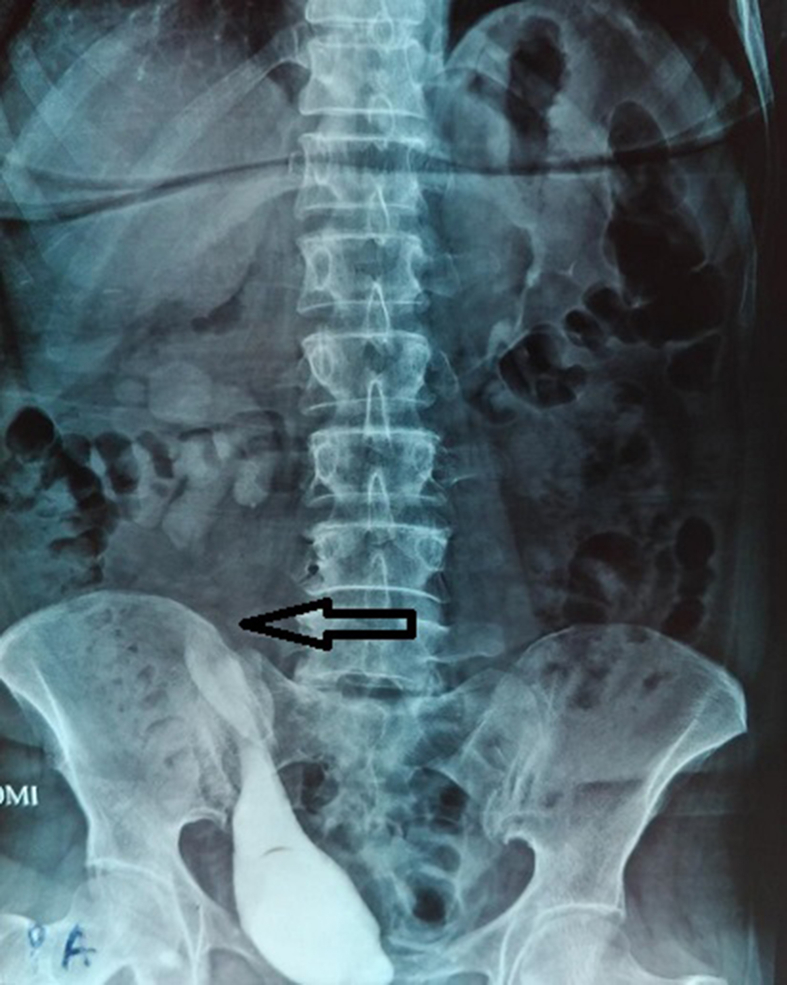
Intravenous Pyelogram (IVP) after calicovesicostomy surgery shows the passage of contrast through the anastomosis site.

In the patient's 4-month follow-up, IVP was performed in which shows the passage of contrast through the anastomosis site (Fig. 3).

## 2 Discussion

Calicovesicostomy is a relatively uncommon surgical technique in which the bladder is anastomosed to the lower calyx of the kidney and used when there is no suitable ureteral tissue. This technique is better performed on large bladders. Usually, the calicostomy at the most dependent part of the hydronephrotic kidney is anastomosed to the vesicostomy at the bladder dome. The volume of the bladder should be at least 300 cc.^3^

This technique reduces the risk of damage to the pelvis and renal arteries and allows urine to enter the bladder directly from the kidneys and allows endoscopic interventions in the future.^4^

Disadvantages of this method include vesicocalyceal reflux, which leads to the risk of pyelonephritis. Therefore, the sterility of urine is very important in these patients. Also, these patients will experience urinary reflux to the kidneys and its complications if there is any obstruction in the outlet of the bladder, such as urethral strictures or enlarged prostate.^5^

According to the mentioned points, patients with this surgery need close follow-up and should be evaluated periodically by urine test and ultrasound.

Because this operation is not common and requires the skill of the surgeon and is usually not the first choice in treating patients, few studies have been done in this field. More studies are needed in this area.

## 3 Conclusion

Calicovesicostomy can be a suitable alternative way in patients with major ureteral damage and can prevent nephrectomy in these patients. This procedure requires the skill of the surgeon and careful follow-up of the patient. Due to vesicoureteral reflux, follow-up of patients and keeping their urine sterile is very important.

## Ethics

Patient informed consent was obtained to publish her information. The patient's private information remained confidential with the researchers.

## Financial support and sponsership

None.

## Roles

Farzad Allameh: Conceptualization, Methodology, Visualization.

Seyyed Ali Hojjati: Supervision, Validation, Writing- Original draft preparation.

Saba Faraji: Writing- Reviewing and Editing.

Amirhossein Eslami: Data curation.

Maryam Garousi: Software, Investigation.

## Declaration of competing interest

The authors declare no conflicts of interest in this work.
